# Laparoscopic simple prostatectomy vs bipolar plasma enucleation of the prostate in large benign prostatic hyperplasia: a two-center 3-year comparison

**DOI:** 10.1007/s00345-020-03512-5

**Published:** 2020-11-11

**Authors:** Riccardo Lombardo, Anton Zarraonandia Andraca, Cristina Plaza Alonso, Juan Andres González-Dacal, Higinio Rodríguez Núñez, Aaron Barreiro Mallo, Barbara Cristina Gentile, Giorgia Tema, Luca Albanesi, Luca Mavilla, Valeria Baldassarri, Cosimo De Nunzio, Andrea Tubaro, Manuel Ruibal Moldes, Roberto Giulianelli

**Affiliations:** 1 Nuova Villa Claudia, Rome, Italy; 2 Complejo Hospitalario de Pontevedra Hospital Montecelo, Pontevedra, Spain; 3 Azienda Ospedaliera Sant’Andrea Roma, ‘Sapienza’ University, Rome, Italy; 4 Department of Urology, University “La Sapienza”, Rome, Italy

**Keywords:** BPH, Laparoscopic simple prostatectomy, BTUEP, LUTS, Prostatic disease

## Abstract

**Purpose:**

To compare surgery outcomes and safety of button bipolar enucleation of the prostate vs laparoscopic simple prostatectomy in patients with large prostates (> 80 g) in a two-center cohort study.

**Methods:**

All patients with lower urinary tract symptoms due to benign prostatic enlargement (Prostate volume > 80 cc) undergoing button bipolar enucleation of the prostate (BTUEP) or laparoscopic simple prostatectomy (LSP) in two centers were enrolled. Data on clinical history, physical examination, urinary symptoms, uroflowmetry and prostate volume were collected at 0, 1, 3 6, 12, 24 and 36 months. Early and long-term complications were recorded.

**Results:**

Overall, 296 patients were enrolled. Out of them, 167/296 (56%) performed a LSP and 129/296 (44%) performed a BTUEP. In terms of efficacy both procedures showed durable results at three years with a reintervention rate of 8% in the LSP group and of 5% in the BTUEP group. In terms of safety, BTUEP and LSP presented similar safety profiles with a 9% of transfusion rate and no major complications.

**Conclusion:**

LSP and BTUEP are safe and effective in treating large-volume adenomas with durable results at three years when performed in experienced centers.

## Introduction

Transurethral resection of the prostate (TURP) is considered the standard procedure for men with prostates between 30 and 80 mL [1, 2]. In patients with larger prostates (> 80 cc), open prostatectomy (OP), holmium laser enucleation (HoLep) and bipolar enucleation (BTUEP) of the prostate represent the gold standard procedures for the management of patients with benign prostatic obstruction (BPO). The management of large adenomas is always challenging and nowadays, the chosen technique often lies on availability and surgeon preference.

According to the latest EAU guidelines, the available techniques (OP; HoLep and bipolar enucleation) in the management of large adenomas (> 80 cc) may be considered equal in terms of short- and long-term functional outcomes [2]. The main limitation of OP is the high morbidity of the procedure, specially due to the high blood transfusion rate (7–14%) [3, 4]. HoLep is mainly limited by the steep learning curve, availability and postoperative transient urinary incontinence; however, it is superior to OP in terms of bleeding [5]. As well, BTUEP has a similar safety profile when compared to HoLep. Advantages of BTUEP include the widely available instrumentation and lower costs [6, 7]. Finally, durability of endoscopic techniques over OP is still unclear.

The exact role of minimally invasive simple prostatectomy (MISP) is still unknown. MISP includes laparoscopic simple prostatectomy (LSP) and robot-assisted simple prostatectomy (RASP) [8, 9].The technique for LSP was first described in 2002 [10], while the first RASP was reported in 2008. According to the latest EAU guidelines, studies are needed to compare the efficacy, safety and hospitalization times of MISP and both OP and endoscopic procedures.

With this knowledge in mind, the aim of our study was to evaluate differences between BTUEP and LSP in terms of efficacy, quality of life (Qol) and perioperative complications.

## Methods

A consecutive series of patients who underwent LSP in Pontevedra Hospital and BTUEP in Nuova Villa Claudia Hospital, between May 2012 and December 2015, were included in the study. Before surgery, all patients signed a dedicated informed consent and approved the use of their data for research purposes. Data were prospectively collected and an internal review board approved the retrospective analysis of the data set. All the procedures were performed in accordance with the Declaration of Helsinki. Any patient with prostate volume (PV) < 80 cc, incomplete data, a prior history of prostatic or urethral surgery, urethral stricture, neuro-vesical dysfunction and/or prostate cancer was excluded from the study. Pontevedra hospital performed only LSP procedures; while NVC clinic, only BTUEP procedures.

Patients with bothersome LUTS were offered surgery if preoperative IPSS ≥ 12 points and/or QoL ≥ 4 and/or maximal urinary flow rate (Qmax) < 15 mL/s and/or post-void residual urine volume (PVR) > 50 mL and/or not-responding to medical therapy and/or not willing to undergo medical therapy [11].

Clinical data, including age, BMI, hemoglobin levels, International Prostate Symptom Score (IPSS), QoL score, prostate-specific antigen (PSA), PV and PVR, were collected.

All operations were performed by expert surgeons.

Primary endpoint of the study was to evaluate medium-term efficacy in terms of symptoms, urinary flow and quality of life (Qol) of BTUEP vs LSP in patients with large prostates and lower urinary tract symptoms due to BPO. Secondary endpoints include safety and medium-term complications.

### Operative techniques

#### LSP

LSP was exclusively performed in Pontevedra hospital. Patient lies in a modified supine position under general anesthesia. Trocars are positioned (Hasson over the umbilicus and then 1 of 12 mm and 3 of 5 mm). A transversal incision is performed on the anterior wall of the prostate capsule for 3–4 cm to identify the prostate adenoma. We proceed with a blunt dissection in the avascular plane of the adenoma laterally and posteriorly. If a third lobe is identified, a dissection of the bladder mucosa is performed followed by a dissection of the posterior plane of the prostate to reach the apex. Finally, the adenoma is detached at the verumontanum level with cold scissors. Hemostasis is controlled using bipolar or monopolar electrocoagulation for minor vessels and two trans-capsular stiches for the pedicles. The trigonization of the prostatic fossa is performed using 2 or 3 stitches between the sacral lip of the bladder neck and posterior surgical capsule. A catheter is introduced. The prostatic capsule is reconstructed with running V-lock suture. The catheter is left inside for 5 days [12].

#### BTUEP

BTUEP was exclusively performed in Nuova Villa Claudia clinic. We performed an en bloc enucleation of the adenoma with an Olympus Surg-Master UES-40 bipolar generator, OES-Pro bipolar resectoscope, continuous-flow saline irrigation and ‘button’ type vapo-resection electrodes (Olympus Europe, Hamburg, Germany). Enucleation begins at the apex of the prostate just laterally to the verumontanum. The mucosa is vaporized and the plane between the adenoma and the capsule is identified. Thereafter, an incision is performed between the median and the left lobe until reaching the bladder neck at 5o’clock. The apical incision at 5 and 7 o’clock is extended with a latero-lateral movement and a disto-proximal compression of the surgeon on the button that creates progressive pedunculation plus tissue vaporization of the left adenoma. Coming back to the initial left apical incision, the mucosa is horizontally incised above the verumontanum, reaching the apex of the right lobe. After the incision of the mucosa within the cleft on the right side of the verumontanum, enucleation of the median and right lobes is carried out exactly as described for the left lobe from 5 to 9 o’clock, joining circumferentially its already detached superior part and the bladder neck. The en bloc enucleated adenoma is now completely isolated in one piece, but still fixed to the bladder neck. At this point, the pedunculated structures of the adenoma are resected with a loop. Meticulous coagulation of the prostatic loggia is then performed [13].

Intraoperative outcomes measured were operative time, weight of resected prostatic tissue and histological features. Perioperative outcomes were duration of bladder irrigation, time to catheter removal, hospital stay and postoperative hemoglobin levels (on day 1). Operative time in BTUEP patients was calculated as time between endoscope insertion to catheter positioning. Operative time in LSP was calculated as first trocar positioning to skin closure.

During the follow-up patients underwent uroflowmetry, I.P.S.S. score, QoL score, PSA and PVR at 1, 3, 6, 12, 24 and 36 months. Both centers followed the patients with the same follow-up schedule.

Perioperative complications were graded according to Clavien Dindo classification as reported in previous experiences by our group [1]. Continence was defined as ‘no-pads’.

Urethral stricture, bladder outlet obstruction (BOO), residual adenoma and postoperative acute urinary retention, as well as reintervention rate were also recorded.

### Statistical analysis

Statistical analyses were performed using Statistical Package for the Social Sciences (SPSS Inc., Chicago, IL, USA). Continuous variables are presented as mean ± standard deviation and were compared using the Mann- Whitney test. Categorical data (percentages) were compared using the chi-square test or Fisher’s exact probability test. Differences from baseline were evaluated using the Wilcoxon test. *p* values < 0.05 were considered statistically significant. Post hoc power calculation was performed for primary outcomes (IPSS, Qmax and QoL) according to Levine et al. using an alpha value of 0,05 resulting in a power > 80% [14].

## Results

Overall, 320 patients were evaluated and out of them, 296 patients presented complete data for the objectives of the study, 167/296 (54%) underwent LSP and 129/296 (46%) underwent BTUEP. At baseline patients undergoing BTUEP were older (73,05 ± 7.48 vs 69,05 ± 7,80; *p* = 0,001) and presented lower prostate volumes (94,80 ± 14.99 vs 108,1 ± 38.98; *p* = 0,001). Baseline characteristics and intraoperative and perioperative data are described in Tables 1 and 2. Overall, volume of tissue retrieved and postoperative Hb were similar in both groups (*p* > 0.05). The BTUEP procedure required significantly shorter operative time than the LSP procedure with a weighted mean difference (WMD) of—33 min (*p* < 0.05) as well as shorter catheterization time with a WMD of—4.38 days (*p* < 0.001). Conversely, postoperative bladder irrigation time (WMD—0.74 days, *p* < 0.001) were significantly shorter in the LSP than in the BTUEP group.

**Table 1:** Baseline characteristics of both groups

	LSP (167 pts)	BTUEP (129 pts)	*p*
Age (years)	69,05 ± 7,80	73.05 ± 7.48	0.001
Hb (g/dl)	14,4 ± 1.41	13.57 ± 1.88	0.225
BMI (kg/m^2^)	27.81 ± 2.8	28.09 ± 2.63	0.689
I.P.S.S	21,00 ± 3,46	23.15 ± 5.65	0.356
Qmax (mL/s)	9,53 ± 4,2	7.07 ± 3,78	0.568
Qol	2.1 ± 0.53	2.39 ± 0.70	0.856
PVR (mL)	85 ± 26.01	85.29 ± 26.13	0.625
TRUS (cc)	108,1 ± 38.98	94. 80 ± 14.99	0.001
PSA (ng/mL)	7.86 ± 5,6	6.25 ± 3.44	0.253

Data are presented as mean ± standard deviation

*IPSS* International Prostate Symptom Score, *BMI* Body Mass Index, *Qmax* maximun flow rate, *PVR* post-voided residual volume, *IIEF* International Index of Erectile Function, *PSA* prostate-specific antigen, *Qol* quality of life

**Table 2:** Peri and postoperative characteristics in both groups

	LSP	BTUEP	*p*
Surgery time (minutes)	130,04 ± 42,2	97.02 ± 25.90	0.001
Volume of tissue retrieve (grams)	60.25 ± 16.9	59.36 ± 17.45	0.876
Percentage of prostate reduction (%)	52,3 ± 15,2	59,5 ± 19,3	0.212
Post-operative Hb (g/dl)	12,34 ± 6,75	10.99 ± 1.27	0.275
Bladder irrigation (days)	0,93 ± 0,47	1,67 ± 0,63	0.001
Catheterization time (days)	6,9 ± 5,15	2,52 ± 0,67	0.001
Hospital Stay (days)	3,3 ± 0,66	3,21 ± 0,63	0.821

Data are presented as mean ± standard deviation

Perioperative complications according to Clavien–Dindo are shown in Table 3. In terms of Grade I complications, patients undergoing BTUEP presented haematuria with clots removal more frequently when compared to LSP (7% vs 2%, *p* < 0,05). In terms of Grade II complications, there were no differences in terms of transfusion rates and AUR after catheter removal. As well, Grade III complications were comparable in both groups with reintervention rates ranging between 2 and 5%. There were no grade IV and V complications.

**Table 3:** Complications according to the Clavien classification system

Complications	LSP (167pts)	BTUEP (129 pts)	*p*
Grade I			
Transient Hematuria	12 (7,1)	2 (1,55)	0.021
UTI	4 (2)	5 (4)	0,249
Acute urinary retention	11 (6,5)	5 (4,1)	0.301
Transient urinary incontinence	3 (2)	2 (1,5)	0,778
Total	30 (18)	14 (11)	0,133
Grade II			
Transfusions	15 (9)	12(9)	0.898
Grade III			
Reintervention (%)	3 (1,7)	6 (4,65)	0.156
Grade IV	0	0	
Grade V	0	0	

Table 4 describes functional outcomes at 1, 3, 6, 12, 24 and 36 months. At each time point, the LSP group presented statistically significant improvements when compared to baseline in urinary symptoms (Range of mean IPSS improvement: 17,3–20,6), quality of life (Range of mean QoL improvement: 2–3), urinary flow (Range of mean Qmax improvement: 10–16 mL/s), PSA levels (Range of mean PSA reduction: 3,6–3,8) and residual urine ( Range of mean PVR reduction: 49–66 cc). As well at each time point, the BTUEP group presented statistically significant improvements when compared to baseline in urinary symptoms ( Range of mean IPSS improvement: 15,5–18,1), quality of life (Range of mean QoL improvement: 2–3), urinary flow (Range of mean Qmax improvement: 7–15 mL/s), PSA levels (Range of mean PSA reduction: 5,6–5,7) and residual urine ( Range of mean PVR reduction: 49–66 cc),

**Table 4:** Functional outcomes after LSP and BTUEP

	LSP group	BTUEP group	*p*
IPSS			
Baseline	21,00 ± 3,46	23.15 ± 5.65	0.356
1 month	5.5 ± 4.4	5.8 ± 3.41	0.882
3 months	4.2 ± 3.16	4.35 ± 3.1	0.326
6 months	3.03 ± 2.56	3.06 ± 1.29	0.453
12 months	3.02 ± 4.14	2.68 ± 0.98	0.523
24 months	2,90 ± 0.62	2.66 ± 1.01	0.325
36 months	2,56 ± 1.43	2.62 ± 0.78	0.423
Qol			
Baseline	2.1 ± 0.53	2.39 ± 0.70	0.856
1 month	5.13 ± 1.45	4.02 ± 1.11	0.856
3 months	5.14 ± 1.55	4.23 ± 1.15	0.754
6 months	4.26 ± 0.91	5.06 ± 0.86	0.956
12 months	4.44 ± 0.84	5.25 ± 0.63	0.965
24 months	4.50 ± 0.82	5.10 ± 0.67	0.754
36 months	4.36 ± 0.73	5.26 ± 0.63	0.954
Qmax			
Baseline	9,53 ± 4,2	7.07 ± 3,78	0.568
1 month	17.4 ± 10.25	17.41 ± 5.75	0.854
3 months	24,8 ± 10,7	20.7 ± 7.36	0.656
6 months	25.72 ± 9.56	21.7 ± 7.22	0.864
12 months	22.62 ± 8.82	23.14 ± 7.25	0.532
24 months	22.73 ± 8,98	22.18 ± 7.13	0.678
36 months	22.21 ± 7.39	22.88 ± 7.04	0.436
PVR			
Baseline	85 ± 26.01	85.29 ± 26.13	0.625
1 month	18.8 ± 17.86	35.2 ± 23.86	0.754
3 months	38.2 ± 15.02	28.2 ± 17.06	0.954
6 months	32.9 ± 13.17	17.75 ± 9.86	0.365
12 months	33.24 ± 13.26	18.6 ± 9.86	0.105
24 months	32.69 ± 12.2	18.8 ± 9.86	0.111
36 months	35.69 ± 11.5	17.8 ± 8.28	0.062
PSA			
Baseline	7.86 ± 5,6	6.25 ± 3.44	0.253
6 months	2,15 ± 0.81	2.38 ± 0.74	0.656
12 months	2,20 ± 0.72	2.39 ± 0.76	0.402
24 months	2,07 ± 0.62	2.56 ± 0.56	0.545
36 months	2,22 ± 0.61	2.37 ± 0.73	0.312

Data are presented as mean ± standard deviation

*IPSS* International Prostate Symptom Score, *BMI* Body Mass Index, *Qmax* maximun flow rate, *PVR* post-voided residual volume, *PSA* prostate-specific antigen, *Qol* quality of life, *BTUEP* bipolar transurethral enucleation of the prostate

As well, at each time point (1, 3, 6, 12, 24 and 36 months), there were no statistically significant differences between groups in terms of IPSS, QoL. Qmax, PSA values and PVR improvements (*p* > 0,05) (Table 4).

Overall, 250/296 (85%) patients completed the 3-year follow-up period (Table 5). During this period, 20 patients were diagnosed of cancer, 20 patients needed a reintervention and 6 patients died of non-urological conditions. Reintervention rate was 4% in the LSP group (4 cases of bladder–neck contracture and 3 urethral strictures) and 5% in BTUEP group (4 bladder–neck contracture and 2 urethral strictures). All the reinterventions occurred in the first year of follow-up.

**Table 5:** Number of patients available for analysis

	BTUEP group	LSP group
Baseline	129	167
1 month	129	167
3 months	123	159
6 months	120	157
12 months	115	150
24 months	107	147
36 months	105	145

## Discussion

The present study compares for the first time BTUEP and LSP for the treatment of large adenomas in patients with BPO. According to our results, both techniques are to be considered equal in terms of functional outcomes. LSP showed longer operative times and longer catheterization times when compared to BTUEP. However, irrigation time was shorter in the LSP group. Both techniques presented good safety profiles with similar transfusion and reintervention rates. Our results are in line with the peer-reviewed literature [15, 16].

The goal of prostate surgery for BPH is to remove the adenoma while minimizing the damage to surrounding structures. According to the latest EAU guidelines, in prostates larger than 80 cc, surgeons should choose between OP, BTUEP or HoLEP [2].

In the past years, LSP has been evaluated by several authors. According to our results, LSP improves symptoms and quality of life at three years (mean IPSS scores: 2,5 ± 1,4; mean Qol score of 4,4 ± 0,7). As well, we observed significant improvements in Qmax and PVR (mean Qmax: 21 ± 7.39 mL/s; mean PVR of 35.69 ± 11.5 mL). Our results are in line with the largest multi-center outcome analysis of the minimally invasive approaches to simple prostatectomy by Autorino et al. The study evaluated 1330 MISP between 2000 and 2014. Of these, 487 (36.6%) were robotic and 843 (63.4%) were pure laparoscopic. They observed significant improvement in functional outcomes at 12-month follow-up [12, 17]. Post-operative median Qmax and median IPSS were 22 (IQR: 20–26) mL/s and 5 (IQR:3–5), respectively, in the laparoscopic group versus 25 (IQR:20–33) mL/s and 7 (IQR:4–9), respectively, in the robotic group.

In our study, we evaluated two minimally invasive techniques. The concept of minimally invasive is still a matter of debate in the literature. Laparoscopy may be considered minimally invasive specially if compared with open prostatectomy; however, it still requires general anesthesia, a Trendelenburg position as well as long catheterization time [13]. It is important to underline that the differences in catheterization time clearly depend on surgical protocol. More specifically, patients on the LSP group routinely remove catheter on day 5, while patients on the BTUEP group on day 2. Probably endoscopic procedures are less invasive when compared to laparoscopy, considering that patient has no need of general anesthesia and has shorter catheterization time. However, in very large prostates (> 200 cc), endoscopy may be challenging and time consuming [18]. In our experience, both techniques showed good functional outcomes and safety profiles at three years. Standing to these results both techniques may be proposed to the patient, which should be aware of the pros and cons of each procedure to make an informed decision [19].

We observed a good safety profile for both techniques; however, some difference were recorded. BTUEP showed higher rates of clots removal when compared to LSP, which has the advantage of a direct coagulation of the whole prostatic loggia. Interestingly, LSP and BTUEP presented similar transfusion rates (9%) and reintervention rates (2–5%). In the BTUEP group, transfusion rate was higher than reported in the literature. A possible explanation for this difference is that our series include old patients with low preoperative Hb levels as well as patients with prostates larger than 140 cc. Conversely, the transfusion rate in the LSP group is slightly lower than the available literature [20]. In our cohort patients, some patients needed reintervention (4–6%). All these patients were evaluated initially with a TRUS to diagnose residual adenoma, with cystoscopy to diagnose urethral stricture and with an urodynamic investigation to confirm obstruction. In both groups, all patients were managed endoscopically. The results are in line with the available evidence on HoLEP [21] on bipolar electrosurgical enucleation of the prostate (BEEP) and on MISP [22, 23].

Secondary endpoints of the study include operative time, length of hospitalization and catheterization period. According to our results, BTUEP showed a more favorable profile compared to LSP considering the mean operative times (130,04 ± 42,2 min vs 97.02 ± 25.90 min, *p* = 0,001) and mean catheterization period (6,9 ± 5,1 vs 2,52 ± 0,67, *p* = 0,001). The longer catheterization depends on the different operative technique considering that LSP requires to open the prostatic capsule with subsequent suturing; however, most of the patients may go home on day 2/3 with the catheter considering that by that time urine is usually clear. Our results are in line with the available evidence comparing these two procedures [24–26].

Although not evaluated in this study, technical aspects, learning curve and costs are important issues when choosing the best surgical option. BTUEP and LSP can be considered easy techniques for surgeons already trained.

In terms of costs, considering that hospital stay is similar, it is assumable that LSP is more expensive than BTUEP in terms of material costs; however, dedicated studies should assess this issue.

Although some differences between different procedures exist, all of them seem equally effective in treating large-volume adenomas. In our study, we have the merit of comparing two techniques performed in highly experienced centers. In each center, a consecutive series of patients undergoing BTUEP (Nuova Villa Claudia) and LSP (Pontevedra Hospital) were enrolled. Although this could be considered a possible limitation, both techniques are considered challenging procedures and so far we preferred to plan the study in centers with a long-lasting experience with these techniques. Our results clearly depend on our population and on patient selection and our results cannot be extended to other centers with no or minimal experience with these techniques. However, the present study is the only available evidence comparing these two techniques in highly experienced centers.

This study presents some limitations. First, the retrospective fashion of the study is a major limitation; however, to overcome this limitation, both databases are prospectively maintained database which were thereafter analyzed retrospectively.

Another important limitation of our study is the fact that all the surgeries were performed by highly experienced endoscopic and laparoscopic surgeons and therefore, the results cannot be extended to all centers. Moreover, our results clearly depend on the enrolled population and cannot be extended to other populations. The lack of urodynamic studies may be considered another limitation. However, most of the studies on endoscopic surgery do not include urodynamic studies [27]. Furthermore, according to latest EAU guidelines, the use of invasive urodynamics studies should be considered only in selected patients which were not included in our study. As well the lack of data on pain management and time to work may be considered a limitation.

We agree that more than one study is necessary to prove a hypothesis. Notwithstanding all these limitations, this study is the only available study comparing LSP and BTUEP outcomes and safety at three years.

## Conclusion

LSP showed similar outcomes and safety at three years when compared to BTUEP in terms of symptoms, urinary flow, Qol, PVR and PSA. However, LSP presented a longer operative, bladder irrigation and catheterization time. Our study opens new insights on the role of BTUEP and LSP for the management of large prostates in patients with LUTS due to BPH. Further studies should clarify the role of these techniques, include cost analysis evaluation and define the best patient profile for both procedures. Theoretically, a tertiary referral center and a BPH clinic should include several surgical options including BTUEP and LSP to tailor the best procedure according to the patient’s characteristics and expectations.
